# Personality predictors of dementia diagnosis and neuropathological burden: An individual participant data meta‐analysis

**DOI:** 10.1002/alz.13523

**Published:** 2023-11-29

**Authors:** Emorie D. Beck, Tomiko Yoneda, Bryan D. James, David A. Bennett, Jason Hassenstab, Mindy J. Katz, Richard B. Lipton, John Morris, Daniel K. Mroczek, Eileen K. Graham

**Affiliations:** ^1^ Department of Medical Social Sciences Northwestern University Feinberg School of Medicine Chicago Illinois USA; ^2^ Department of Psychology University of California Davis Davis California USA; ^3^ Rush Alzheimer's Disease Center Department of Internal Medicine Rush University Medical Center Chicago Illinois USA; ^4^ Department of Neurology Rush Alzheimer's Disease Center Rush University Medical Center Chicago Illinois USA; ^5^ Department of Neurology Washington University School of Medicine St. Louis Missouri USA; ^6^ Department of Neurology Albert Einstein College of Medicine Bronx New York USA; ^7^ Department of Psychology Northwestern University Weinberg College of Arts & Sciences Evanston Illinois USA

**Keywords:** agreeableness, arteriosclerosis, Braak stage, CERAD, cerebral amyloid angiopathy, cerebral atherosclerosis, extraversion, gross cerebral infarcts, gross cerebral microinfarcts, hippocampal sclerosis, individual participant data meta‐analysis, Lewy body disease, openness, positive affect, satisfaction with life, TDP‐43

## Abstract

**INTRODUCTION:**

The extent to which the Big Five personality traits and subjective well‐being (SWB) are discriminatory predictors of clinical manifestation of dementia versus dementia‐related neuropathology is unclear.

**METHODS:**

Using data from eight independent studies (*N*_total_= 44,531; *N*_dementia_= 1703; baseline *M*_age_= 49 to 81 years, 26 to 61% female; M_follow‐up_ range = 3.53 to 21.00 years), Bayesian multilevel models tested whether personality traits and SWB differentially predicted neuropsychological and neuropathological characteristics of dementia.

**RESULTS:**

Synthesized and individual study results indicate that high neuroticism and negative affect and low conscientiousness, extraversion, and positive affect were associated with increased risk of long‐term dementia diagnosis. There were no consistent associations with neuropathology.

**DISCUSSION:**

This multistudy project provides robust, conceptually replicated and extended evidence that psychosocial factors are strong predictors of dementia diagnosis but not consistently associated with neuropathology at autopsy.

**Highlights:**

- N(+), C(−), E(−), PA(−), and NA(+) were associated with incident diagnosis.
- Results were consistent despite self‐report versus clinical diagnosis of dementia.
- Psychological factors were not associated with neuropathology at autopsy.
- Individuals with higher conscientiousness and no diagnosis had less neuropathology.
- High C individuals may withstand neuropathology for longer before death.

## 1 BACKGROUND

The incidence of dementia due to neurodegenerative diseases has increased substantially over the past half‐century along with increases in life expectancy,^1^ contributing to an expansive economic burden and disability.^2^, ^3^ Identifying modifiable risk factors that influence individual differences in cognitive aging processes is critical to researchers, policymakers, and the public. While research suggests that the Big Five personality traits and subjective well‐being (SWB) are associated with dementia diagnosis,^4^, ^5^, ^6^ limited research has examined traits or SWB as predictors of underlying dementia neuropathology. Drawing data from eight independent studies (ie, a multistudy approach), the current study investigated whether the Big Five (extraversion, agreeableness, conscientiousness, neuroticism, and openness to experience) and SWB (life satisfaction, positive affect, and negative affect) differentially predict dementia diagnoses and neuropathological burden. This approach also permitted opportunities to explore evidence linking these psychological constructs to the cognitive resilience theoretical model.

Several different neuropathologies cause dementia; the most well‐known type of dementia, Alzheimer's disease (AD), is defined by amyloid beta (Aβ) peptides and tau neurofibrillary tangles (NFTs), which subsequently results in loss of neuronal cells. Although AD is the leading cause of dementia, there are other types of dementia (eg, vascular, frontotemporal, Lewy body), and the majority of dementia cases are due to mixed pathologies.^7^ A large body of research demonstrates a disconnect between the degree of pathology in a person's brain and whether that neuropathology manifests clinically as cognitive impairment^8^, ^9^, ^10^; approximately one‐third of cognitively *unimpaired* older adults aged 75+ years have sufficient Aβ and NFTs to meet AD criteria. Numerous systematic reviews and meta‐analyses indicate that physical, social, and cognitive engagement contributes to healthier cognitive aging.^13^, ^14^, ^15^, ^16^, ^17^, ^18^ The Big Five personality traits capture consistent patterns in physical, social, and cognitive engagement and can be conceptualized as higher‐order predictors of factors contributing to cognitive aging. Indeed, the existing literature documents associations between cognitive functioning and dementia diagnosis with the Big Five, particularly neuroticism and conscientiousness.^4^, ^5^, ^6^,^19^, ^20^, ^21^, ^22^, ^23^, ^24^

Multiple pathways linking personality traits and dementia have been proposed^25^; two likely accounts theorize that traits may (1) act as predispositions that subsequently influence brain health and/or (2) influence cognitive performance in the presence of neuropathological burden. For instance, individuals high in conscientiousness demonstrate healthier behavioral, emotional, and cognitive tendencies across the lifespan, which protect against development of neuropathology (ie, contributing to brain maintenance) and/or assist in maintaining better cognitive performance despite the development of neuropathology (ie, cognitive *resilience*). Evidence supporting the predisposition theory finds links between traits and cortical amyloid deposition, tau pathology, and smaller brain volume assessed by in vivo biomarkers, brain imaging, and autopsy.^26^, ^27^, ^28^, ^29^, ^30^ Evidence supporting the cognitive resilience model finds that individuals high in conscientiousness or low in neuroticism are less likely to develop clinical dementia despite neuropathology at autopsy.^20^, ^31^, ^32^

**RESEARCH IN CONTEXT**

1. **Systematic review**: We reviewed the literature within Web of Science, PubMed, and EBSCOhost electronic databases. Limited research has examined the relationships between personality or well‐being and neuropathology, though several publications examine the associations between personality or well‐being and dementia diagnosis. No research has systematically investigated the links between personality, well‐being, clinical manifestation of dementia, and neuropathology all together or using an individual participants meta‐analytic approach. We appropriately cite relevant research.
2. **Interpretation**: Our findings, based on 44,531 participants from eight longitudinal samples spanning three continents and five countries, highlight clear differences in the associations between these psychosocial factors (ie, personality traits, well‐being) and clinical versus neuropathological manifestations of dementia. Conscientiousness, extraversion, and positive affect may improve, while neuroticism and negative affect may impede, performance on neuropsychological tests, leading to differential risk of receiving a dementia diagnosis.
3. **Future directions**: Future research should prospectively investigate similar associations using in vivo markers of dementia.

Research on personality and dementia rarely assesses neuropathological markers of neurodegenerative disease, making the distinction between these models impossible to test. Our multistudy approach permits evaluation of the replicability and robustness of prospective associations between traits and dementia diagnosis using large samples that span decades and continents, as well as exploration of the processes linking traits to the diagnosis of dementia and neuropathology. Specifically, we not only test whether personality traits are separately associated with clinical diagnoses and neuropathology (predisposition theory) but also test whether personality traits moderate the association between clinical diagnoses and neuropathology (cognitive resilience theory). Finally, additional psychological factors, such as SWB, may contribute to cognitive aging processes. SWB can be conceptualized as a tripartite construct (life satisfaction, negative affect, and positive affect).^33^ Some evidence suggests that certain aspects of well‐being are associated with cognitive resilience^34^ and that satisfaction with life is protective against dementia diagnoses,^35^, ^36^ but this literature is small and newly emerging. We address this gap in the literature by investigating SWB as an antecedent of incident dementia diagnoses and neuropathological burden.

## 2 METHOD

This study makes two primary contributions to the literature. It (1) examines aspects of the processes that may underlie the association between psychological factors (the Big Five and SWB), incident dementia diagnosis, and *post mortem* neuropathology and (2) integrates across multiple samples simultaneously to better estimate robustness and generalizability using a one‐stage individual participant data meta‐analysis (IPD‐MA). First, while alternative or additional processes may underlie these associations,^25^, ^31^, ^37^ our design permitted exploration of foundational associations between both the disease burden itself (neuropathology) and the clinical manifestation (dementia risk). Second, prior research in this area is typically based on single studies or meta‐analyses of published studies. The use of individual participant data from multiple studies has a number of advantages, including the ability to directly control for key covariates and moderators and generally not being subject to choices made by researchers who worked with the raw data.^38^ With IPD‐MA, we are able to make identical data cleaning, harmonization, and analytic choices across studies. Thus, rather than statistically correcting for these different choices as in traditional meta‐analyses, IPD‐MA enables us to clearly and directly compare effect sizes across samples. Further, IPD‐MA is also not subject to publication bias.

Investigating associations among personality traits, SWB, clinical dementia, and neuropathology in a multistudy format permits evaluation of associations across samples, measures, and time while preserving important heterogeneity across studies. Systematic investigation of the prospective relationships between personality or SWB with neuropsychological and neuropathological markers of dementia may provide important information regarding the mechanisms underlying these associations and the timing in which they unfold, potentially informing the development of interventions and screening assessments. Importantly, personality and well‐being assessments can be administered quickly and cost‐effectively, whereas neuropsychological batteries and biomarker collection can be time‐consuming, costly, and stress‐inducing for patients.^39^, ^40^ Integrating personality and well‐being assessments in clinical settings earlier in the lifespan can help to identify long‐term risk for a number of chronic illnesses and offer unique pathways for interventions before symptom onset.^41^, ^42^

We test three primary research questions. First, we ask whether the Big Five personality traits and aspects of SWB are associated with dementia diagnoses and neuropathology at autopsy. Second, we ask whether sociodemographic and baseline cognitive health factors (age, gender, education, and global cognition) moderate associations between the Big Five/SWB and diagnoses/neuropathology. Finally, we ask whether the Big Five and SWB moderate associations between dementia diagnoses and neuropathology at autopsy. This study was preregistered on the Open Science Framework (https://osf.io/fmjv3). In addition, all code, model objects, figures, and tables are available in the online materials on the OSF (https://osf.io/dzty7/) and GitHub (https://github.com/emoriebeck/personality‐dementia‐neuropath/tree/master/results). Finally, rendered results are available as a standalone web page on GitHub (https://emoriebeck.github.io/personality‐dementia‐neuropath/) and in an online R Shiny web app (https://emoriebeck.shinyapps.io/personality‐dementia‐neuropath/).

### 2.1 Participants

Participants included 44,531 individuals from eight longitudinal samples, spanning two continents and four countries. We chose samples based on prior work examining personality predictors of cognitive decline, dementia diagnoses, and neuropathology).^20^, ^28^, ^37^ From these we identified six samples (Washington University School of Medicine Memory and Aging Project [WUSM‐MAP], Rush Memory and Aging Project [Rush‐MAP], Religious Orders Study [ROS], Einstein Aging Study [EAS], Baltimore Longitudinal Study of Aging [BLSA], and Health and Retirement Study [HRS]). One (BLSA) was eliminated because we were not granted access to the data. We identified three additional samples (German Soeconomic Panel Study [GSOEP], Longitudinal Internet Studies for the Social Sciences [LISS], and Swedish Adoption / Twin Study of Aging [SATSA]) that had personality trait measures and dementia diagnoses. Across samples, we used the latest data release, and participants were included in all models in which they had requisite data (ie, participants within samples vary across combinations of personality, SWB, covariates, and moderators when necessary). Sample descriptions are available in the online materials.

### 2.2 Measures

To conduct IPD‐MA, variables across studies must be harmonized, which involves pulling, recoding, and including measures that have exact (ie, measured and coded identically) or conceptual (ie, measured and coded differently, but recoded to the same scale) mappings across samples. A more in‐depth discussion of this process was previously documented.^43^ In the present study, because measures were not identical across samples, we used conceptual harmonization, which is described in detail in subsequent sections. Descriptive statistics of all conceptually harmonized variables for each sample are presented in Table 1. Zero‐order correlations among measures within samples are presented in the online materials and web app.

**TABLE 1:** Descriptive statistics of all harmonized measures across samples.

Variable	Rush‐MAP	ROS	WUSM‐MAP	EAS	GSOEP	HRS	LISS	SATSA
**Big Five personality traits**								
Measurement tool	NEO‐FFI	NEO‐FFI	NEO‐PI R	IPIP NEO	BFI‐S	MIDI	IPIP50	Eysenck
Extraversion	5.50 (1.69)	5.17 (1.65)	5.73 (1.67)	5.59 (1.75)	6.40 (1.88)	7.33 (1.85)	5.64 (1.62)	6.33 (0.74)
Agreeableness		5.33 (1.40)	6.37 (1.62)	6.73 (1.87)	7.45 (1.63)	8.42 (1.59)	6.68 (1.48)	7.23 (1.17)
Conscientiousness	6.25 (1.57)	6.27 (1.39)	6.31 (1.79)	6.95 (1.73)	8.21 (1.55)	7.85 (1.61)	6.34 (1.49)	7.05 (1.35)
Neuroticism	3.65 (1.68)	4.59 (1.61)	3.94 (1.91)	3.56 (2.08)	4.92 (2.04)	3.51 (2.06)	6.03 (1.69)	6.53 (1.35)
Openness to experience		5.50 (1.51)	5.60 (59)	5.85 (2.03)	5.88 (2.01)	6.44 (1.87)	5.98 (1.34)	4.84 (1.34)
**Subjective well‐being**								
Positive affect	8.46 (1.82)	8.20 (3.86)			6.22 (2.15)	6.56 (1.93)	5.94 (1.67)	7.47 (2.12)
Negative affect	1.06 (1.30)				3.63 (1.98)	1.92 (1.81)	1.75 (1.76)	2.14 (2.29)
Satisfaction with life	7.39 (1.78)	6.75 (2.20)			7.00 (1.76)	6.08 (2.34)	6.56 (1.46)	5.75 (2.87)
**Clinical dementia diagnosis**								
Valid *N*	4283	3374	1478	813	31,072	14,001	6543	2002
Clinical dementia	444 (22.17%)	428 (30.77%)	232 (27.39%)	44 (5.67%)	180 (1.03%)	1130 (7.98%)	20 (0.31%)	163 (8.14%)
Mean dementia follow‐up (years)	5.48 (4.14)	9.56 (6.61)	15.59 (22.11)	2.70 (2.92)	11.20 (0.48)	6.76 (2.26)	5.19 (4.43)	21.00 (0.00)
**Neuropathology**								
Valid *N*	1721	1525	627	37				
Mean neuropathology follow‐up (years)	6.16 (3.62)	10.46 (6.04)	14.83 (22.50)	7.29 (3.52)				
Braak stage	3.77 (1.20)	3.58 (1.25)	4.01 (1.66)	3.12 (1.06)				
CERAD	2.16 (1.12)	2.22 (1.13)	2.46 (0.81)					
Lewy body disease	177 (23.14%)	177 (23.44%)	58 (38.67%)	4 (11.76%)				
Gross cerebral infarcts	298 (37.91%)	271 (34.70%)	158 (100.00%)					
Gross cerebral microinfarcts	260 (33.08%)	245 (31.37%)	27 (17.09%)					
Cerebral atherosclerosis	1.13 (0.77)	1.24 (0.78)	1.29 (0.76)					
Cerebral amyloid angiopathy	1.29 (0.95)	1.29 (0.97)	1.27 (0.88)					
Arteriosclerosis	1.03 (0.91)	0.99 (0.96)	1.42 (0.70)					
Hippocampal sclerosis	69 (9.77%)	56 (7.64%)	3 (3.66%)	4 (11.76%)				
TDP‐43	428 (54.31%)	353 (49.79%)	27 (31.40%)					
**Covariates**								
Age	81.12 (6.96)	76.53 (7.24)	71.37 (10.39)	79.57 (5.39)	48.51 (16.93)	67.93 (10.45)	46.54 (15.94)	59.76 (14.02)
Gender (% female)	532 (25.93%)	417 (28.64%)	474 (56.63%)	476 (61.34%)	9166 (52.48%)	8438 (59.56%)	3568 (54.53%)	1177 (58.79%
Education	14.87 (3.15)	18.25 (3.48)	15.71 (2.95)	14.46 (3.39)	11.60 (2.52)	12.61 (3.08)	12.60 (4.19)	10.32 (1.82)
Global cognition	6.55 (1.02)	6.43 (0.98)	3.92 (1.29)	4.98 (0.81)		7.18 (1.58)	3.08 (2.81)	5.49 (1.35)
Body mass index (BMI)	27.03 (5.02)	27.32 (5.39)		28.28 (5.09)	25.61 (4.35)	27.66 (4.42)	25.42 (7.82)	25.50 (3.80)
Self‐rated health				3.93 (2.94)	6.75 (2.30)	6.36 (2.78)	5.39 (1.89)	2.16 (2.79)
Smokes	870 (42.52%)	285 (19.57%)	97 (11.58%)	423 (54.51%)	5617 (33.97%)	7935 (56.01%)	4009 (61.97%)	998 (49.85%)
Alcohol use	596 (36.01%)	362 (27.57%)	44 (5.25%)	714 (92.01%)	9800 (58.31%)	7523 (53.10%)	5953 (92.02%)	1334 (67.89%)
Race (% Other)	361 (17.59%)	224 (15.38%)	126 (15.05%)	343 (44.20%)		5074 (35.82%)		
Stroke	174 (9.26%)	96 (6.95%)	17 (2.03%)	34 (4.38%)	0 (0.00%)	1410 (9.95%)	52 (0.81%)	40 (2.06%)
Cancer	676 (32.96%)	444 (30.49%)	56 (6.68%)	136 (17.62%)	0 (0.00%)	2500 (17.65%)	104 (1.62%)	77 (3.98%)
Diabetes	275 (13.41%)	193 (13.26%)	75 (8.95%)	141 (18.24%)	0 (0.00%)	3376 (23.83%)	305 (4.76%)	124 (6.38%)
Heart problems	249 (12.15%)	147 (10.10%)	70 (8.35%)	170 (21.91%)		4367 (30.83%)	252 (3.93%)	667 (33.91%)

*Note*: Sample sizes are based on the number of participants with at least one personality trait or well‐being measurement occasion and at least one valid dementia diagnosis or neuropathology indicator. Age, education, gender, smoking, alcohol, BMI, chronic conditions, and cognition were assessed at the first baseline personality assessment. See supplementary materials for full details on cognitive measures and scoring across samples. No. cases (%) values represent binary indicators (eg, No. women [proportion of women] for gender), while continuous indicators are presented as M (SD).

#### 2.2.1 Psychosocial characteristics: The Big Five and SWB

Complete information on the scales used for each measure across samples is in Table S1, and which measures are available across samples is documented in Table S2.^1^ Many measures are on different scales, so all psychosocial indicators were transformed to *P*ercentages *O*f the *M*aximum *P*ossible score (POMP).^44^ *z*‐transformations have a mean of zero and unit variance, which can be useful for interpreting effect sizes in standard deviation or correlational terms. However, when the underlying distribution is non‐normal, such interpretations can be less clear. POMP, in contrast, allows for interpretation in relative percentiles. To aid convergence, we deviate from traditional POMP scoring and multiply the ratio by 10:

(1) $$\begin{array}{c} POMP=\frac{observed-min}{max-min}\times 10. \end{array}$$

#### 2.2.2 Dementia diagnoses

The measurement of dementia diagnoses varied across samples, including clinician assessments (Rush‐MAP, ROS, WUSM‐MAP, EAS, SATSA), participant‐reported clinical dementia diagnoses (ie, received a dementia diagnosis from a doctor ever or in the last year; HRS, LISS, GSOEP), and probability of dementia diagnosis based on cognitive testing (HRS). Each was recoded such that 0 = no clinical dementia and 1 = dementia diagnosis.

#### 2.2.3 Neuropathology

We identified 10 indicators of *post mortem* neuropathology from four samples: Braak stage (a measure of AD severity capturing the degree and diffuseness of NFTs; 0 to 6), CERAD (a measure of AD capturing the presence of neuritic plaques; 1 to 4, reverse coded), Lewy body disease (0 = no, 1 = yes), gross cerebral infarcts (0 = none, 1 = one or more), gross cerebral microinfarcts (0 = none, 1 = one or more), cerebral atherosclerosis (0 to 3), cerebral amyloid angiopathy (0 to 3), arteriosclerosis (0 to 3), hippocampal sclerosis (0 = none, 1 = present), and TAR DNA‐binding protein‐43 (TDP‐43; 0 = none or amygdala only, 1 = beyond amygdala). More details on the background and measurement of each can be found in the online materials. Three samples (WUSM‐MAP, Rush‐MAP, and ROS) used standard National Alzheimer's Coordinating Center (NACC) autopsy checklists and data preparation. For EAS, indicators were transformed or omitted as necessary to ensure exact comparability with NACC checklists (Table 1).

#### 2.2.4 Participant‐level covariates and moderators

Covariates included age (years, centered at 60), gender (0 = male, 1 = female), and education (years, centered at 12). Cognitive function was POMP scored (POMP scores derived from similar tests across identical broad cognitive domains, see supplemental material for more details; 0 to 10). More details on each of the covariates can be found in the codebook in the online materials and rendered results in the web app. In what follows, we focus on results from the adjusted models. The full results for all models are available online.

### 2.3 Analytic plan

To test whether personality and SWB were prospectively associated with dementia diagnoses and neuropathology, we fit a series of Bayesian multilevel models (with a random intercept and a random slope for personality traits and SWB) predicting diagnosis and neuropathology (11) from each predictor (8) separately across each of the sets of covariates.^2^ Such multilevel models are an example of one‐stage IPD‐MA in which sample‐level and meta‐analytic effects are estimated in a single model.^3^ In these models, participants’ observations (Level 1) were nested within study (Level 2). Covariates were included at Level 1 but were not modeled as random effects. The basic form of the unadjusted model is as follows:

$$Y_{ij}=\beta_{0j}+\beta_{1j}*P_{ij}+\varepsilon_{ij},$$

$$\beta_{0j}=\gamma_{00}+u_{0j},$$

$$\beta_{1j}=\gamma_{10}+u_{1j},$$

$$e_{ij}∼N\left(0,\sigma^{2}\right),$$

(2) $$\begin{array}{c} u_{0j}\\ u_{1j} \end{array}∼N\left(\begin{array}{cc} \tau_{00}^{2} & \tau_{10}\\ \tau_{10} & \tau_{11}^{2} \end{array}\right)\;,$$

where *i* indicates individual *i* in sample *j* and *P* indicates levels on a personality trait or well‐being characteristic (in POMP scores). The key terms are $\gamma_{10}$, which captures the meta‐analytic association between personality trait/well‐being levels and each outcome; $u_{1j}$, which captures the sample‐specific deviation from the overall estimate; and $\beta_{1j}$, which is the linear combination of the overall estimate and the deviation that captures the sample‐specific estimate. We used regularizing priors (*t*‐distributed) for all fixed effects, half‐Cauchy priors for all variances, and LKJ priors for all correlations.

Binary outcomes (dementia diagnosis, Lewy body disease, gross cerebral infarcts, gross cerebral microinfarcts, hippocampal sclerosis, and TDP‐43) were modeled using logistic regression models with a logit link, while continuous outcomes (Braak stage, CERAD, cerebral atherosclerosis, cerebral amyloid angiopathy, and arteriosclerosis) were modeled using linear regression with a Gaussian link. The results of logistic regression models are presented as odds ratios (ORs; exponentiated log odds), while results of linear regression models are presented as non‐standardized estimates (ie, change in neuropathology associated with a 10% difference in personality trait or SWB levels).

Next, to test (1) whether age, gender, education, and cognitive function moderate the association between levels of personality characteristics and dementia diagnoses and neuropathology and (2) whether personality and SWB moderate the relationship between dementia diagnoses and neuropathology, we added a main effect and interaction for each moderator separately at Level 1 and included them as random slopes. The basic form of the model is as follows:

$$Y_{ij}=\beta_{0j}+\beta_{1j}\times P_{ij}+\beta_{2j}\times M_{ij}+\beta_{3j}\times P_{ij}\times M_{ij}+\varepsilon_{ij},$$

$$\beta_{0j}=\gamma_{00}+u_{0j},$$

$$\beta_{1j}=\gamma_{10}+u_{1j},$$

$$\beta_{2j}=\gamma_{20}+u_{2j},$$

$$\beta_{3j}=\gamma_{30}+u_{3j},$$

$$e_{j}∼N\left(0,\sigma^{2}\right),$$

(3) $$\begin{array}{c} u_{0j}\\ u_{1j}\\ u_{2j}\\ u_{3j} \end{array}∼N\left(\begin{array}{cccc} \tau_{00}^{2} & \tau_{01} & \tau_{02} & \tau_{03}\\ \tau_{10} & \tau_{11}^{2} & \tau_{12} & \tau_{13}\\ \tau_{20} & \tau_{21} & \tau_{22}^{2} & \tau_{23}\\ \tau_{30} & \tau_{31} & \tau_{32} & \tau_{33}^{2} \end{array}\right),$$

where *M* indicates the level of the moderator for person *i* in sample *j*, and the new key terms are $\gamma_{20}$ and $\beta_{2j}$, which capture the main effect of the moderator across all samples (ie, the meta‐analytic effect) and for each sample separately, respectively, and $\gamma_{30}$ and $\beta_{3j}$, which capture the interaction between personality trait/well‐being levels and moderator levels across all samples (ie, the meta‐analytic effect) and for each sample separately, respectively. Errors and random terms are assumed normal with variances $\sigma^{2}$ (residual variance) and $\tau^{2}$ (random effect variance).

**FIGURE 1:**
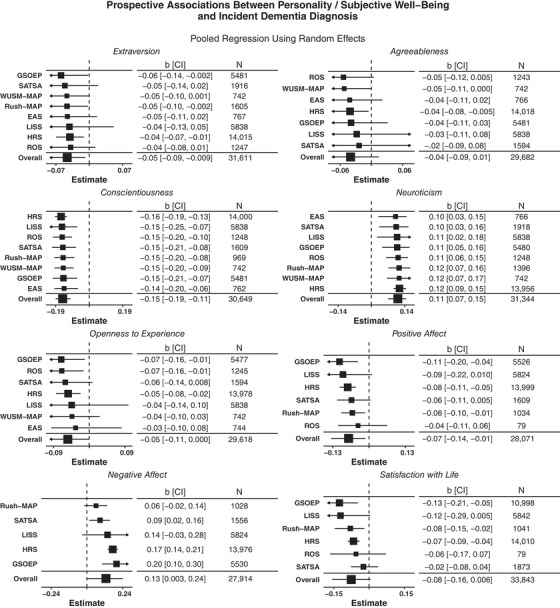
Forest plots of prospective associations between eight personality characteristics and incident dementia diagnoses. Point estimates (OR) represent the exponentiated mean of the posterior distribution. Interval estimates represent the 95% credible intervals (CI) of the exponentiated posterior distribution of the overall estimate and linear combinations of the overall estimate and random effects for sample‐specific estimates. *N* indicates the sample size with complete data for each predictor, outcome, and set of covariates.

Due to missing data, sample sizes vary across models. In each model, we used all participants with complete data for the indicators included in the model. Sample sizes used in each model are included in all forest plots of results across studies (eg, Figure 1). Because sample sizes reported in Table 1 indicate the number of participants with any valid data for any combination of psychosocial characteristics (ie, personality traits and SWB), the sample sizes reported in Table 1 may not align with the number of observations in any given model.

### 2.4 Deviations from preregistration

Although this study was preregistered to avoid making any analytic choices after hypothesizing, a small number of unforeseen challenges and questions led to slight deviations from the preregistration. First, we originally identified eight covariates (age, gender, education, cognitive function, marital status, self‐rated health, chronic conditions [diabetes, stroke, cancer, respiratory disease], smoking, and alcohol use) and planned to test unadjusted and fully adjusted models. Due to sampling differences in which and when measures were collected, only one sample (HRS) could be fully adjusted. Thus, we identified which covariates were both ever collected and collected at or before baseline personality measurement in all samples and focused on these for the main analyses.^4^ Second, in our preregistered analyses, we did not investigate whether the timing of diagnoses or death relative to the assessment of personality and well‐being impacted our findings. Thus, we elected to additionally adjust for this interval in all models. Third, we elected to add an additional research question (whether personality traits and SWB moderate dementia diagnosis–neuropathology associations) after preregistering our hypotheses and analytic plan. Our goal was to allow us to disentangle evidence for traits as predispositions for brain maintenance and/or traits contributing to cognitive resilience across the samples. Finally, as noted previously, we preregistered the use of data from the BLSA but were not granted access to the data.

### 2.5 Prior publications of the same samples

Data from the samples used in this study were previously published. Table S3 provides detailed information on prior publications using similar data, summaries of findings, and summaries of key differences between each of those studies and the present study. Two samples (GSOEP, LISS) have no prior publications of personality traits and dementia diagnoses. Among other previously published studies using the same samples, the current study differs in key ways that make the re‐analysis of data from these samples value added. In some cases, we add additional years (sometimes decades) of data (HRS,^46^ ROS,^6^ Rush‐MAP,^47^). In others, we use different inclusion criteria or subsets of the sample (WUSM‐MAP;^31^ EAS,^48^, ^49^ SATSA,^20^). In all cases but Duchek et al.,^31^ prior investigation used Cox Proportional Hazards Models (eg, Wilson et al.,^6^ Terracciano et al.^46^), tested whether personality traits moderated cognitive decline and dementia conversion,^20^ or tested whether personality trait change predicted dementia diagnoses.^49^ See Table S3 in the online materials and web app for additional information on prior uses of the samples and comparisons with the present study.

## 3 RESULTS

### 3.1 Association of personality and well‐being with clinical dementia

First, we examined prospective associations between the Big Five/SWB and dementia diagnoses. As seen in Figure 1 (forest plots with sample‐specific estimates) and Table 2 (meta‐analytic associations), neuroticism (+), conscientiousness (−), extraversion (−), positive affect (−), and negative affect (+) were associated with risk of dementia across studies. We conducted a total of 63 hypothesis tests, including the meta‐analytic term, across eight different models (one for each psychosocial characteristic). Of these, 37 (58.7%) were significant. Both neuroticism and conscientiousness were associated with incident dementia diagnoses in every sample and overall, extraversion in three out of eight samples and overall, negative affect in three out of five samples and overall, positive affect (−) in three out of six samples and overall, and satisfaction with life (−) in three of six samples but not overall. Across all samples and psychological characteristics, estimates tended to be in the same direction, but of slightly different magnitudes.

**TABLE 2:** Meta‐analytic estimates of adjusted personality–dementia diagnosis and neuropathology associations.

	Incident dementia diagnosis	Braak stage	CERAD	Lewy body disease	Gross cerebral infarcts	Gross cerebral microinfarcts	Cerebral atherosclerosis	Cerebral amyloid angiopathy	Arteriosclerosis	Hippocampal sclerosis	TDP‐43
Trait	OR [CI]	b [CI]	b [CI]	OR [CI]	OR [CI]	OR [CI]	b [CI]	b [CI]	b [CI]	OR [CI]	OR [CI]
**None**											
Extraversion	0.95 [0.92, 0.99]	−0.01 [−0.28, 0.15]	−0.03 [−0.10, 0.03]	1.01 [0.91, 1.14]	1.03 [0.70, 1.99]	0.96 [0.83, 1.08]	0.01 [−0.06, 0.09]	0.02 [−0.04, 0.07]	−0.01 [−0.07, 0.09]	0.96 [0.82, 1.16]	1.00 [0.79, 1.22]
Agreeableness	0.96 [0.92, 1.01]	−0.02 [−0.13, 0.09]	−0.02 [−0.29, 0.22]	0.99 [0.76, 1.37]	1.15 [0.42, 3.24]	1.09 [0.61, 1.74]	−0.01 [−0.19, 0.16]	0.00 [−0.32, 0.20]	−0.00 [−0.38, 0.17]	1.00 [0.61, 1.62]	0.98 [0.68, 1.50]
Conscientiousness	0.86 [0.83, 0.90]	−0.05 [−0.14, 0.02]	0.03 [−0.07, 0.14]	0.99 [0.81, 1.32]	1.01 [0.64, 1.92]	1.05 [0.83, 1.35]	0.00 [−0.05, 0.07]	0.02 [−0.06, 0.13]	−0.02 [−0.09, 0.04]	1.05 [0.85, 1.35]	1.01 [0.87, 1.21]
Neuroticism	1.12 [1.07, 1.16]	0.04 [−0.11, 0.31]	−0.01 [−0.11, 0.09]	1.00 [0.77, 1.16]	1.13 [0.67, 2.31]	0.99 [0.85, 1.13]	0.02 [−0.05, 0.09]	0.01 [−0.07, 0.08]	0.01 [−0.07, 0.10]	1.03 [0.87, 1.21]	1.03 [0.88, 1.20]
Openness to experience	0.95 [0.90, 1.00]	−0.02 [−0.13, 0.08]	−0.03 [−0.21, 0.15]	1.03 [0.77, 1.32]	1.09 [0.37, 3.15]	0.94 [0.69, 1.43]	−0.03 [−0.26, 0.12]	−0.06 [−0.19, 0.15]	−0.05 [−0.42, 0.25]	1.03 [0.75, 1.50]	1.02 [0.76, 1.39]
Positive affect	0.93 [0.87, 0.99]	−0.05 [−0.36, 0.18]	0.00 [−0.26, 0.17]	1.01 [0.67, 1.45]	1.07 [0.80, 1.83]	1.02 [0.74, 1.45]	0.01 [−0.15, 0.15]	0.03 [−0.12, 0.19]	−0.01 [−0.18, 0.18]	1.09 [0.72, 1.70]	1.05 [0.71, 1.54]
Negative affect	1.14 [1.00, 1.27]	−0.11 [−1.65, 1.09]	−0.09 [−1.26, 1.01]	1.04 [0.33, 3.79]	1.02 [0.26, 4.16]	1.14 [0.24, 4.75]	0.00 [−1.30, 1.12]	−0.01 [−1.28, 1.19]	−0.08 [−1.32, 1.09]	1.16 [0.26, 8.64]	1.03 [0.26, 4.26]
Satisfaction with life	0.93 [0.85, 1.01]	0.01 [−0.29, 0.31]	−0.00 [−0.26, 0.23]	1.05 [0.78, 1.47]	1.15 [0.69, 2.22]	1.10 [0.74, 2.08]	0.02 [−0.20, 0.20]	0.001 [−0.35, 0.35]	0.00 [−0.28, 0.28]	0.79 [0.40, 2.17]	1.22 [0.85, 1.90]
Age											
Extraversion	1.00 [1.00, 1.01]	0.001 [−0.01, 0.01]	−0.000 [−0.01, 0.01]	1.00 [0.99, 1.01]	1.01 [0.93, 1.09]	1.00 [0.99, 1.02]	−0.000 [−0.00, 0.00]	−0.000 [−0.01, 0.01]	0.000 [−0.01, 0.01]	1.01 [0.99, 1.02]	0.99 [0.98, 1.01]
Agreeableness	1.00 [1.00, 1.01]	−0.00 [−0.02, 0.01]	−0.01 [−0.06, 0.03]	1.00 [0.95, 1.04]	0.95 [0.79, 1.29]	1.00 [0.94, 1.07]	−0.00 [−0.06, 0.03]	−0.01 [−0.04, 0.04]	−0.00 [−0.05, 0.04]	0.97 [0.93, 1.01]	1.00 [0.92, 1.07]
Conscientiousness	1.01 [1.00, 1.01]	−0.001 [−0.01, 0.01]	0.001 [−0.01, 0.01]	1.00 [0.99, 1.02]	1.01 [0.94, 1.08]	1.00 [0.99, 1.02]	0.001 [−0.00, 0.01]	−0.00 [−0.01, 0.00]	0.000 [−0.01, 0.01]	1.01 [0.99, 1.03]	1.00 [0.99, 1.01]
Neuroticism	1.00 [0.99, 1.00]	0.00 [−0.01, 0.01]	−0.00 [−0.01, 0.01]	1.00 [0.99, 1.01]	1.02 [0.95, 1.08]	1.00 [0.99, 1.01]	0.001 [−0.01, 0.01]	0.00 [−0.00, 0.01]	0.00 [−0.02, 0.01]	1.00 [0.98, 1.02]	1.00 [0.98, 1.02]
Openness to experience	1.00 [1.00, 1.01]	0.01 [−0.02, 0.06]	−0.01 [−0.06, 0.02]	0.99 [0.97, 1.02]	0.98 [0.68, 1.23]	1.00 [0.95, 1.06]	0.00 [−0.03, 0.03]	−0.00 [−0.04, 0.02]	−0.00 [−0.04, 0.02]	1.02 [0.98, 1.06]	0.99 [0.93, 1.06]
Positive affect	1.00 [0.99, 1.01]	−0.01 [−0.05, 0.03]	−0.00 [−0.07, 0.04]	0.99 [0.92, 1.06]	1.01 [0.96, 1.08]	0.99 [0.92, 1.06]	0.00 [−0.03, 0.04]	−0.00 [−0.04, 0.03]	−0.00 [−0.04, 0.03]	1.01 [0.92, 1.12]	1.05 [0.94, 1.16]
Negative affect	1.00 [0.99, 1.00]	−0.01 [−0.68, 0.64]	0.11 [−0.32, 0.41]	1.07 [0.76, 1.74]	1.00 [0.55, 1.69]	1.01 [0.63, 1.73]	−0.03 [−0.38, 0.35]	−0.02 [−0.50, 0.35]	−0.02 [−0.38, 0.24]	1.07 [0.70, 2.04]	0.95 [0.63, 1.75]
Satisfaction with life	1.00 [1.00, 1.01]	−0.00 [−0.04, 0.03]	−0.00 [−0.04, 0.04]	1.00 [0.94, 1.05]	1.02 [0.98, 1.06]	0.99 [0.93, 1.06]	0.00 [−0.02, 0.03]	−0.00 [−0.04, 0.05]	0.001 [−0.03, 0.04]	1.04 [0.92, 1.13]	0.99 [0.94, 1.06]
Gender											
Extraversion	0.95 [0.89, 1.01]	0.01 [−0.10, 0.11]	−0.15 [−0.56, 0.18]	1.08 [0.75, 1.58]	1.04 [0.60, 2.25]	0.95 [0.70, 1.32]	−0.04 [−0.14, 0.05]	−0.01 [−0.17, 0.13]	−0.07 [−0.21, 0.09]	0.94 [0.71, 1.24]	0.95 [0.73, 1.23]
Agreeableness	0.99 [0.92, 1.08]	0.09 [−0.16, 0.26]	−0.04 [−0.63, 0.54]	1.12 [0.68, 1.99]	1.09 [0.21, 5.54]	0.92 [0.40, 2.36]	−0.05 [−0.59, 0.38]	−0.02 [−0.52, 0.43]	0.01 [−0.48, 0.94]	0.85 [0.49, 1.65]	0.89 [0.49, 1.79]
Conscientiousness	0.99 [0.93, 1.07]	−0.05 [−0.23, 0.06]	−0.02 [−0.16, 0.19]	0.96 [0.51, 1.24]	1.23 [0.65, 2.85]	0.98 [0.71, 1.31]	0.01 [−0.11, 0.13]	−0.000 [−0.14, 0.13]	−0.01 [−0.19, 0.18]	1.12 [0.77, 1.64]	0.91 [0.71, 1.17]
Neuroticism	1.01 [0.94, 1.07]	−0.01 [−0.13, 0.10]	−0.01 [−0.21, 0.23]	0.92 [0.65, 1.30]	0.96 [0.53, 2.03]	0.95 [0.73, 1.25]	−0.01 [−0.17, 0.14]	0.00 [−0.21, 0.15]	−0.24 [−0.85, 0.12]	1.03 [0.75, 1.41]	0.97 [0.76, 1.23]
Openness to experience	1.00 [0.92, 1.11]	−0.03 [−0.21, 0.19]	−0.01 [−0.55, 0.45]	1.18 [0.73, 2.29]	1.07 [0.31, 5.22]	0.91 [0.37, 1.88]	−0.03 [−0.32, 0.31]	0.001 [−0.34, 0.40]	−0.04 [−0.48, 0.54]	0.75 [0.43, 1.58]	1.07 [0.57, 2.04]
Positive affect	1.03 [0.94, 1.13]	−0.01 [−0.54, 0.51]	−0.001 [−0.70, 0.53]	0.85 [0.21, 2.65]	0.67 [0.11, 3.03]	1.52 [0.44, 7.49]	−0.05 [−0.55, 0.38]	−0.02 [−0.67, 0.53]	−0.01 [−0.48, 0.59]	0.81 [0.23, 2.59]	0.67 [0.19, 1.90]
Negative affect	0.92 [0.73, 1.06]	−0.03 [−2.04, 2.54]	−0.04 [−2.10, 1.95]	0.94 [0.12, 8.11]	0.90 [0.18, 5.48]	0.75 [0.08, 5.91]	0.18 [−1.91, 2.35]	−0.05 [−2.08, 1.87]	0.06 [−1.93, 1.64]	1.36 [0.12, 10.75]	1.21 [0.15, 9.81]
Satisfaction with life	0.98 [0.91, 1.08]	0.05 [−0.77, 0.68]	−0.17 [−1.12, 0.76]	0.94 [0.26, 2.57]	0.80 [0.13, 3.06]	1.49 [0.38, 7.58]	0.02 [−0.61, 0.52]	0.04 [−0.52, 0.51]	0.06 [−0.42, 0.57]	1.19 [0.25, 5.23]	0.82 [0.17, 2.59]
Education											
Extraversion	1.00 [0.99, 1.01]	−0.01 [−0.02, 0.01]	0.01 [−0.01, 0.03]	0.98 [0.90, 1.04]	1.01 [0.91, 1.15]	1.01 [0.96, 1.07]	−0.001 [−0.02, 0.02]	−0.001 [−0.02, 0.02]	0.00 [−0.02, 0.02]	1.00 [0.96, 1.05]	0.99 [0.96, 1.03]
Agreeableness	1.00 [0.99, 1.01]	−0.01 [−0.05, 0.02]	0.01 [−0.07, 0.11]	1.00 [0.94, 1.06]	0.97 [0.65, 1.39]	1.02 [0.84, 1.24]	0.00 [−0.06, 0.05]	−0.01 [−0.09, 0.06]	−0.02 [−0.08, 0.06]	0.96 [0.83, 1.09]	1.01 [0.90, 1.13]
Conscientiousness	0.99 [0.98, 1.00]	0.00 [−0.01, 0.02]	0.01 [−0.02, 0.03]	0.98 [0.91, 1.05]	1.04 [0.93, 1.29]	1.00 [0.96, 1.03]	−0.000 [−0.02, 0.01]	0.000 [−0.02, 0.02]	0.00 [−0.01, 0.02]	0.98 [0.93, 1.04]	0.99 [0.94, 1.02]
Neuroticism	1.00 [0.99, 1.01]	−0.01 [−0.02, 0.01]	−0.000 [−0.02, 0.02]	1.00 [0.96, 1.04]	0.99 [0.88, 1.16]	0.98 [0.88, 1.05]	−0.00 [−0.02, 0.01]	−0.00 [−0.02, 0.02]	−0.01 [−0.03, 0.01]	1.02 [0.96, 1.09]	1.00 [0.96, 1.04]
Openness to experience	1.00 [0.99, 1.02]	−0.01 [−0.04, 0.02]	0.001 [−0.09, 0.08]	1.00 [0.95, 1.08]	0.96 [0.64, 1.29]	0.93 [0.66, 1.18]	−0.01 [−0.06, 0.05]	0.000 [−0.07, 0.08]	−0.001 [−0.07, 0.07]	0.97 [0.87, 1.12]	0.97 [0.89, 1.07]
Positive affect	1.00 [0.99, 1.01]	−0.01 [−0.10, 0.06]	0.001 [−0.12, 0.10]	0.89 [0.57, 1.30]	1.01 [0.86, 1.20]	1.01 [0.89, 1.20]	0.00 [−0.08, 0.10]	0.01 [−0.07, 0.11]	0.04 [−0.08, 0.15]	1.00 [0.83, 1.29]	1.03 [0.84, 1.20]
Negative affect	1.00 [0.98, 1.03]	−0.09 [−1.43, 1.05]	−0.17 [−1.48, 0.98]	1.12 [0.33, 10.09]	0.98 [0.30, 2.95]	1.08 [0.34, 3.63]	−0.02 [−1.10, 0.94]	−0.04 [−1.07, 0.99]	0.03 [−1.08, 1.31]	1.11 [0.36, 4.38]	1.01 [0.37, 2.95]
Satisfaction with life	1.00 [0.99, 1.01]	−0.00 [−0.09, 0.10]	−0.01 [−0.14, 0.09]	0.97 [0.79, 1.24]	1.00 [0.84, 1.20]	1.03 [0.83, 1.37]	−0.000 [−0.13, 0.12]	0.00 [−0.16, 0.16]	0.00 [−0.09, 0.09]	0.99 [0.48, 1.67]	0.99 [0.84, 1.17]
Cognition											
Extraversion	1.00 [0.98, 1.03]	0.00 [−0.04, 0.04]	0.01 [−0.04, 0.05]	0.95 [0.90, 1.00]	1.01 [0.82, 1.26]	0.97 [0.90, 1.03]	−0.01 [−0.03, 0.01]	−0.01 [−0.05, 0.02]	0.00 [−0.03, 0.03]	1.04 [0.95, 1.13]	1.02 [0.95, 1.10]
Agreeableness	1.00 [0.97, 1.05]	0.01 [−0.09, 0.09]	0.02 [−0.08, 0.19]	0.96 [0.86, 1.08]	0.94 [0.59, 1.55]	0.94 [0.81, 1.09]	−0.01 [−0.12, 0.07]	−0.00 [−0.10, 0.11]	−0.01 [−0.12, 0.13]	1.11 [0.93, 1.36]	1.06 [0.84, 1.28]
Conscientiousness	0.99 [0.93, 1.05]	−0.01 [−0.04, 0.02]	0.01 [−0.04, 0.05]	0.95 [0.90, 1.01]	0.97 [0.80, 1.18]	1.00 [0.90, 1.09]	−0.01 [−0.05, 0.03]	0.001 [−0.03, 0.03]	−0.000 [−0.03, 0.03]	1.04 [0.93, 1.17]	0.97 [0.88, 1.04]
Neuroticism	1.01 [0.99, 1.03]	−0.01 [−0.05, 0.03]	0.00 [−0.05, 0.05]	1.01 [0.94, 1.08]	1.01 [0.84, 1.27]	0.98 [0.92, 1.06]	0.02 [−0.01, 0.05]	−0.000 [−0.05, 0.04]	0.02 [−0.03, 0.08]	0.95 [0.86, 1.05]	0.97 [0.91, 1.05]
Openness to experience	0.99 [0.96, 1.04]	0.000 [−0.06, 0.06]	−0.05 [−0.18, 0.14]	0.97 [0.82, 1.08]	0.99 [0.68, 1.45]	0.97 [0.83, 1.19]	−0.04 [−0.26, 0.05]	0.01 [−0.11, 0.08]	−0.01 [−0.09, 0.06]	0.96 [0.81, 1.15]	1.07 [0.88, 1.43]
Positive affect	0.99 [0.96, 1.02]	−0.02 [−0.16, 0.08]	−0.01 [−0.10, 0.07]	1.05 [0.91, 1.22]	1.05 [0.93, 1.21]	1.02 [0.89, 1.15]	0.01 [−0.08, 0.11]	0.02 [−0.08, 0.11]	0.001 [−0.12, 0.10]	1.10 [0.88, 1.38]	1.03 [0.80, 1.34]
Negative affect	1.01 [0.91, 1.17]	−0.03 [−0.74, 0.59]	−0.11 [−0.88, 0.53]	1.09 [0.42, 2.28]	0.99 [0.41, 2.32]	1.01 [0.39, 2.15]	0.03 [−0.65, 0.78]	−0.07 [−0.86, 0.65]	−0.05 [−0.89, 0.72]	1.34 [0.52, 5.63]	0.88 [0.44, 2.31]
Satisfaction with life	1.00 [0.97, 1.04]	−0.03 [−0.18, 0.09]	0.02 [−0.10, 0.16]	1.06 [0.88, 1.33]	1.04 [0.88, 1.24]	1.02 [0.84, 1.30]	−0.000 [−0.11, 0.12]	0.02 [−0.09, 0.13]	0.02 [−0.13, 0.16]	0.91 [0.68, 1.24]	1.01 [0.87, 1.20]

*Note*: Adjusted models Include age, gender, education, smoking status, alcohol use, and prediction interval. Sections of the table preceded by bold text indicate the moderators that were included in the model. Point estimates indicate the median of the posterior distribution, while interval estimates indicate the 95% credible interval (CI) of the posterior. For binary outcomes, these are exponentiated values to transform log odds to odds ratios. Bold values indicate 95% CIs that do not overlap with zero (before exponentiation for binary outcomes).

### 3.2 Association of personality and well‐being with neuropathological burden

Next, we examined associations between personality traits/SWB and 10 neuropathological indicators of dementia at autopsy (Table 2), including a total number of 270 statistical tests (80 meta‐analytic tests and 190 sample‐specific tests). Across all studies, there was no consistent association between psychological characteristics and neuropathology measures. Sample‐specific estimates are presented in the online materials and online web app. Although a small number of sample‐specific estimates (7/190; 3.7%) were significant, this was never the case for more than one study for each psychological characteristic–outcome combination and fell within the expected 5% type‐I error rate, suggesting the possibility of spurious associations.

### 3.3 Moderators of personality/well‐being associations with dementia and neuropathology

**FIGURE 2:**
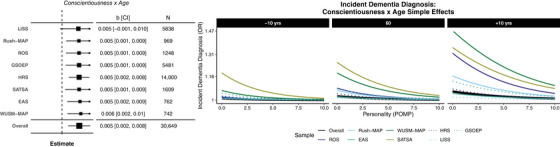
Forest plots of significant overall and sample‐specific estimates of age (in years, centered at 60) and education (in years, centered at 12) moderating the association between Big Five personality characteristics (8) and later dementia diagnosis (left). OR = median exponentiated log odds ratio of the posterior; CI = 95% Bayesian credible interval. Simple effects plots visualizing the relationship between conscientiousness (in POMP units, 0 to 10) and probability of dementia diagnosis (OR) across ages in all samples (overall; thick, black lines) and for each sample separately (shaded and dashed lines).

Table 3 presents the overall estimates of four tested moderators (age, gender, education, baseline cognition) predicting neuropathology and clinical diagnoses. This included a total of 1224 statistical tests, including 352 meta‐analytic tests. Across all outcomes, 43 tests were significant (3.2%). But among these, 18/244 (7.38%) of moderator tests for clinical dementia and 20/2000 (0.92%) of moderator tests for neuropathology were significant. For clinical dementia diagnoses, age moderated the relationship between conscientiousness and dementia diagnosis (see Figure 2 for overall and sample‐specific estimates). Figure 2 illustrates dementia risk across levels of conscientiousness as a function of age (in years, centered at 60) and education (in years, centered at 12; conscientiousness only). There was a stronger protective relationship between both conscientiousness and dementia diagnosis for 70‐year‐old adults than those at 50 or 60 years old.

**TABLE 3:** Meta‐analytic estimates of adjusted dementia diagnosis–neuropathology associations moderated by personality traits and well‐being.

	Braak stage	CERAD	Lewy body disease	Gross cerebral infarcts	Gross cerebral microinfarcts	Cerebral athero‐sclerosis	Cerebral amyloid angiopathy	Arteriosclerosis	Hippocampal sclerosis	TDP‐43
Trait	b [CI]	b [CI]	OR [CI]	OR [CI]	OR [CI]	b [CI]	b [CI]	b [CI]	OR [CI]	OR [CI]
Extraversion		0.04 [−0.09, 0.18]	0.04 [−0.37, 0.61]	1.01 [0.84, 1.23]	1.07 [0.62, 2.65]	1.13 [0.90, 1.40]	0.03 [−0.07, 0.15]	0.04 [−0.06, 0.20]	0.02 [−0.07, 0.12]	1.02 [0.73, 1.41]
Agreeableness		0.05 [−0.15, 0.25]	−0.03 [−0.63, 0.56]	1.11 [0.75, 1.65]	1.08 [0.30, 4.40]	1.01 [0.43, 4.15]	0.05 [−0.37, 0.34]	−0.01 [−0.50, 0.36]	−0.02 [−0.49, 0.43]	0.99 [0.53, 2.01]
Conscientiousness		0.10 [−0.07, 0.26]	−0.05 [−0.29, 0.22]	1.06 [0.84, 1.32]	1.09 [0.65, 2.19]	1.15 [0.89, 1.49]	0.03 [−0.07, 0.15]	0.03 [−0.06, 0.14]	0.00 [−0.12, 0.12]	0.86 [0.57, 1.28]
Neuroticism		−0.07 [−0.21, 0.07]	0.07 [−0.16, 0.32]	1.10 [0.84, 1.40]	1.03 [0.57, 1.89]	0.92 [0.63, 1.45]	−0.03 [−0.14, 0.09]	−0.03 [−0.17, 0.12]	0.001 [−0.13, 0.12]	0.87 [0.62, 1.24]
Openness to experience		0.01 [−0.19, 0.25]	0.03 [−0.79, 0.74]	1.13 [0.85, 1.57]	1.01 [0.26, 3.79]	1.09 [0.58, 2.53]	−0.06 [−0.46, 0.29]	−0.00 [−0.41, 0.48]	0.03 [−0.42, 0.57]	1.24 [0.76, 2.26]
Positive affect		−0.02 [−0.88, 0.46]	−0.03 [−0.57, 0.48]	1.12 [0.67, 2.48]	0.93 [0.56, 1.73]	0.85 [0.44, 2.01]	−0.02 [−0.48, 0.39]	−0.05 [−0.34, 0.27]	0.02 [−0.48, 0.57]	1.12 [0.61, 2.85]
Negative affect		−0.12 [−2.01, 1.59]	−0.31 [−1.64, 1.31]	1.21 [0.22, 10.72]	1.05 [0.19, 6.33]	1.11 [0.14, 8.85]	−0.60 [−2.31, 1.44]	−0.02 [−1.64, 1.57]	−0.02 [−1.88, 1.47]	0.89 [0.15, 6.47]
Satisfaction with life		−0.12 [−1.39, 0.57]	−0.03 [−0.56, 0.39]	1.13 [0.58, 2.08]	0.83 [0.36, 1.73]	0.93 [0.44, 2.52]	−0.01 [−0.44, 0.41]	−0.05 [−0.54, 0.45]	−0.01 [−0.44, 0.39]	1.26 [0.25, 7.53]

*Note*: Adjusted models Include age, gender, education, smoking status, alcohol use, and prediction interval. Point estimates indicate the median of the posterior distribution, while interval estimates indicate the 95% credible interval (CI) of the posterior. For binary outcomes, these are exponentiated values to transform log odds to odds ratios. None of the 95% CIs overlapped with zero (before exponentiation for binary outcomes), indicating no significant meta‐analytic results.

For neuropathology, meta‐analytic estimates suggested no consistently moderated associations between personality/SWB and neuropathology. Forest plots and simple effects plots for all other personality traits, outcomes, and moderators are in the online materials and online web app.

### 3.4 Personality and well‐being moderators of clinical dementia‐neuropathology associations

**FIGURE 3:**
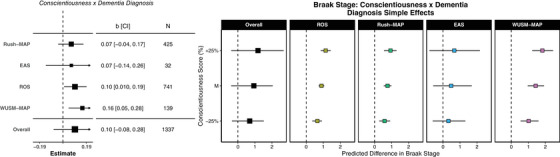
Forest plots of overall and sample‐specific estimates of clinical dementia diagnosis (0 = no, 1 = yes) moderating the association between Big Five personality characteristics (8) and neuropathology indicators at autopsy (left). OR = median exponentiated log odds ratio of posterior; CI = 95% Bayesian credible interval. Simple effects plots visualizing the relationship between conscientiousness (in POMP units, 0 to 10) and neuropathology (in stages 0 to 6 for Braak stage across all samples (overall; thick, black lines) and for each sample separately (shaded lines).

Next, we tested whether personality traits and well‐being moderated the association between neuropathology and clinical dementia diagnoses. If personality traits or well‐being are associated with larger differences in neuropathological burden between those who were or were not diagnosed with dementia, this provides evidence for resilience models (ie, do people higher in conscientiousness have more neuropathology than we would expect based on their diagnosis status than people lower in conscientiousness?). We conducted a total of 275 tests, including 80 meta‐analytic tests, of which six (2.2%) were significant. Conscientiousness moderated the association between Braak stages and clinical dementia (2/4, ROS and WUSM‐MAP; Figure 3). That is, people who were higher (rather than lower) in conscientiousness had different levels of Braak stages than we would expect based on their clinical dementia diagnosis status alone. Examining the marginal means suggests that those who were diagnosed had higher Braak stages overall, as expected. However, across participants who were not diagnosed with dementia during the time in study, individuals who were higher in conscientiousness had lower Braak stages than participants lower in conscientiousness in WUSM‐MAP and ROS. These results suggest that conscientiousness may be protective against development of neuropathology, which is consistent with the *resistance* to neuropathology hypothesis. Figure 3 depicts associations across samples (left) and simple effects plots (right).

## 4 DISCUSSION

The current IPD‐MA investigated whether psychological factors (the Big Five traits and three aspects of SWB) predicted neuropsychological and neurological markers of dementia using a multistudy framework. Replicating and extending prior publications in the same samples by including additional waves of follow‐up (ROS, Rush‐MAP, EAS, HILDA, HRS, WUSM‐MAP) and extending these analyses to new samples (LISS, GSOEP), results indicate robust prospective associations between some psychological factors and incident dementia diagnosis, but not neuropathology. Specifically, neuroticism and negative affect were risk factors for, while conscientiousness, extraversion, and positive affect were protective against dementia diagnosis. Across all analyses, there was directional consistency in estimates across samples (*see forest plots*, Figure 1), which is particularly noteworthy given between‐study differences in sociodemographic and design characteristics (eg, sample size, age at baseline, frequency of occasions, years of follow‐up). Consistent with our preregistered hypotheses, these results replicate and extend evidence that personality traits may assist in early identification and dementia‐care planning strategies, as well as risk stratification for dementia diagnosis. Moreover, our findings provide further support for recommendations to incorporate psychological trait measures into clinical screening or diagnosis criteria.^41^, ^50^, ^51^ Conversely, these psychological factors were not consistently associated with any neuropathology indicators. For example, neuroticism was not directly associated with neuropathology biomarkers, suggesting that individuals higher in neuroticism do not have more neuropathological burden at death, consistent with previous research.^6^, ^52^

Our follow‐up moderation analyses suggested that baseline cognitive function did not consistently moderate associations between personality traits and neuropathology. Further, across synthesized analyses, personality traits did not moderate the associations between dementia diagnoses and neuropathology. These findings are inconsistent with the postulation that particular traits may protect against the development of neuropathology. However, synthesized moderator analyses and some *individual study* results revealed some evidence supporting the cognitive resilience model; specifically, older individuals were more likely to have higher Braak stages, gross cerebral infarcts, cerebral atherosclerosis, cerebral, amyloid angiopathy, arteriosclerosis, hippocampal sclerosis, and TDP‐43, and lower CERAD. As synthesized results suggested that older individuals who were also higher in conscientiousness were less likely to be diagnosed with dementia, high conscientiousness may be protective against dementia diagnosis in the face of possible neuropathology (ie, cognitive resilience). Indeed, individuals higher in conscientiousness who did not receive a clinical diagnosis tended to have a lower Braak stage at autopsy in ROS and WUSM‐MAP. Together, these findings hint at the possibility that conscientiousness is related to cognitive resilience. However, given that this neuropathology finding was only replicated in half of the datasets, results should be interpreted with caution, but they emphasize the need for future research efforts focusing on traits, dementia diagnosis, and Braak stage.

The reliable association between negative affect and dementia diagnosis is a particularly novel contribution to the literature. This finding aligns well with mounting evidence from multiple studies on seemingly remarkable linear associations between emotions rated as integers on Likert‐like scales and a number of consequential outcomes.^53^ Negative affect is characterized by a variety of aversive mood states (eg, anger, anxiety, disgust, guilt, fear)^54^, and, when assessed on several occasions, average negative affect is highly related to neuroticism.^55^ As such, it is unsurprising that both negative affect and neuroticism were positively associated with dementia diagnosis. Similarly to the possible inflammatory pathways underlying the link between neuroticism and dementia,^56^, ^57^, ^58^ research suggests that negative affect is associated with neuroinflammation, particularly for individuals high in Aβ load.^59^ Abnormal immune response and inflammatory processes may cause neural system change, thereby predisposing individuals to depressive symptoms,^60^, ^61^ which are positively associated with high and dysregulated negative affect.^62^ That is, the link between inflammation and psychological factors appears to be bidirectional^63^, ^64^ (eg, depressive symptoms are related to inflammation,^65^ and inflammation may cause depressive symptoms^66^). The current study examined only a single measure of negative affect as a predictor of incident diagnosis and neuropathology; however, intraindividual variability in mood states is typical across the lifespan.^67^ Future research should make use of longitudinal measurement burst designs that assess day‐to‐day negative affect, to examine prospective associations between average levels of and variability in affect in relation to dementia diagnosis and neuropathology.

Finally, our findings provide some evidence that openness to experience, positive affect, and satisfaction with life may be protective against incident dementia diagnosis, though effects were only significant in 42%, 50%, and 50% of studies, respectively. With regard to openness, our findings are consistent with previous research as well as our hypotheses, which reveal mixed associations between openness and aspects of cognition and dementia.^4^, ^5^, ^57^, ^68^ Importantly, openness to experience, which is characterized by cognitive flexibility and engagement,^69^ is the least consistent Big Five trait in cross‐cultural replications and across personality taxonomies.^70^ Given cross‐cultural differences in openness, heterogeneity across our findings may be partially attributed to disparate meanings of openness items across datasets or individuals. Furthermore, openness tends to be associated with cognitive processes, possibly capturing aspects of cognitive functioning^71^; as such, the timing of openness assessment may influence associations with dementia diagnosis (ie, openness assessments in prodromal stages of dementia may lead to lower self‐reported openness in tandem with awareness of cognitive decline). Despite this, we saw inconsistent evidence across studies of openness predicting diagnoses, even when adjusting for the timing of assessments.

A notable strength of the current IPD‐MA was thorough preregistration of the research design, variable harmonization, analytic plan, and hypotheses (https://osf.io/fmjv3). The primary deviations were follow‐up moderator analyses, which provided a better test of whether personality moderates the relationship between level of neuropathological burden and the clinical manifestation of dementia, and adjusting for assessment intervals, which provides more robust evidence that our findings represent truly prospective effects. Furthermore, a substantial strength is our IPD‐MA approach, which permitted estimation of overall robustness of personality and well‐being predictors of dementia and pathology while preserving real and important heterogeneity in prediction across studies. Importantly, estimates were directionally consistent despite between‐study differences in operational definitions of dementia diagnosis (eg, self‐report vs clinical diagnoses), providing support for this harmonization approach. Future research should aim to systematically disentangle and harmonize these measures and their associations with both personality and dementia diagnoses. Finally, given the extensive analyses included within this IPD‐MA, figures depicting results from all analyses are available in the online R Shiny web app (https://emoriebeck.shinyapps.io/personality‐dementia‐neuropath/).

An important limitation of the current study was the limited access to neuropathology markers; half of the samples did not complete autopsies, and all samples with neuropathology markers were US samples. Additionally, the LISS dataset only included 20 dementia cases, limiting our confidence in the power to detect associations between psychosocial factors (personality traits and SWB) and risk of dementia. If we had investigated these research questions in only one dataset, this would have been especially concerning. However, our one‐stage approach is particularly effective for estimating effects when events such as dementia diagnoses are rare. Further, the included studies are not representative with respect to race. Given emerging evidence that dementia and cognitive decline unfold differently for Black and Mexican American populations in the United States,^72^ efforts to understand the role of race are critically important, requiring concerted data collection efforts focused on these historically marginalized groups.

With regard to the analytic approach, the primary goal was to map basic associations between baseline psychological factors with dementia diagnoses and neuropathology at autopsy. However, these are likely dynamic associations that vary over time and will require more nuanced understanding of how personality (and personality changes), cognitive function (and cognitive decline), and neuropathology unfold together, which requires longitudinal modeling and in vivo biomarkers of dementia‐causing diseases, including AD.^30^, ^73^ Future work using a joint modeling approach, in which the association between psychological factors and cognitive functioning trajectories is examined in relation to in vivo and/or autopsy neuropathology markers, may better delineate the mechanisms underlying the links between the Big Five, dementia diagnosis, and neuropathology.

Overall, the current IPD‐MA replicated and extended prior work, providing strong evidence that neuroticism, conscientiousness, and negative affect are associated with dementia diagnoses across samples, measures, and time. The directional consistency in estimates despite between‐study differences in operational definitions of dementia diagnoses emphasizes the practicality of using either self‐report or clinical diagnoses of dementia, contributing to conceptual replication efforts. Further, our results suggest a protective effect of openness to experience, positive affect, and satisfaction with life for incident dementia diagnosis, though effects were less consistent across datasets. Although the Big Five and aspects of SWB were not associated with neuropathology at autopsy, moderator analyses reveal some evidence that these psychological factors may also act as predispositions that influence neuropathology.^20^ Future work is needed to build upon these key findings, focusing on more nuanced, time‐varying questions to determine the temporal ordering of these associations and mechanisms underlying them.

## CONFLICTS OF INTEREST STATEMENT

The content is solely the responsibility of the authors and does not necessarily represent the official views of the funding agencies. There are no conflicts of interest to disclose among any of the contributing authors. Author disclosures are available in the supporting information.

## Supporting information

Supplemental Information.

Supplemental Information.

Supplemental Information.

Supplemental Information.

## Footnotes

1 Some scales, such as neuroticism and negative affect, have both theoretical overlap and overlapping assessment content. We chose to separate these constructs rather than compute global composites for two reasons. First, although there is theoretical overlap between neuroticism and negative affect, there is also critical theoretical divergence. Specifically, negative affect aims to tap levels of negative affective experiences, while neuroticism additionally aims to capture instability and reactivity in negative affective experiences. Second, zero‐order correlations (see online materials) among all psychosocial characteristics indicate that correlations between neuroticism and negative affect (range r 0.35 to 0.59) were not large enough to expect redundant measures. Neuroticism and negative affect are also linked to depressive symptoms, which in turn are associated with risk of dementia. Similar to neuroticism and negative affect, there is theoretical and empirical convergence and divergence. Theoretically and empirically, high negative affect (particularly sadness) is a core depressive symptom (and linked to neuroticism). Yet, depressive symptoms tend not to measure emotional instability (a core aspect of neuroticism) because depression is characterized by more steady negative emotions. Depressive symptoms also capture a number of behavioral symptoms that are weakly or inconsistently associated with neuroticism and negative affect, like fatigue, poor concentration, sleeping problems, and appetite changes.

2 All models were estimated using the R statistical programming language using the brms package in R. In addition, we used the psych, knitr, kableExtra, readxl, haven, broom.mixed, rstan, cowplot, plyr, tidyverse, and furrr packages.

3 There are a variety of statistical tools available for conducting IPD‐MA. These are often grouped into two broad categories: one‐stage approaches in which all samples are included in a single model and two‐stage approaches in which all samples are separately analyzed and their effect sizes then synthesized via meta‐analysis. The advantages and disadvantages of one‐ versus two‐stage IPD‐MA is an important ongoing area of research in many quantitative domains (eg, Riley et al.^45^). Nearly all research to date suggests that one method does not always outperform the other and that in most circumstances the results from the methods should be very similar, particularly for the meta‐analytic estimates that are often the focus of using IPD‐MA (see Riley et al.^45^ for a review). Their differences are typically the result of (1) partial pooling and (2) likelihood estimation. Partial pooling is a feature of multilevel or hierarchical models in which information is shared across clusters (ie, samples in the present study) in order to reduce bias in clusters with small amounts of data and/or with outlying effect sizes (relative to the average). One‐stage approaches also directly estimate the likelihood, while this must be approximated in two‐stage approaches. In this study, we opted for a one‐stage approach because we had some (a) small samples, (b) rare event counts in some samples, (c) and very unbalanced event rates in some samples to take advantage of exact likelihoods and partial pooling. Following comments from a helpful reviewer, we replicated all of our one‐stage models using a two‐stage approach. These results almost exactly replicated both the point and interval estimates of all meta‐analytic associations (both for main effects and moderators). In some cases, there were slight differences in sample‐specific estimates, but these tended to be in the interval estimates more than the point estimates. Such slight differences are expected when using multilevel models with partial pooling that results in shrinkage of estimates. The full two‐stage results can be found in the online materials and web app.

4 We also tested two other sets of covariates (see online materials) and found nearly identical results. First, we tested the most common set of covariates in longitudinal research (age, gender, education). Second, we tested covariates that were included in every sample but one. Marital status, smoking, alcohol use, and chronic conditions (diabetes, stroke, cancer, and respiratory disease) were dichotomized (0 = no, 1 = yes). We also adjusted for self‐rated health (scored within study on Likert‐like scales, operationalized as POMP scores) and cognitive function (POMP scores derived from similar tests across identical broad cognitive domains, see supplemental material for more details; 0‐10). Third, we tested each of the above but additionally included dementia diagnosis as a covariate. Across all models, results were nearly identical, so we report unadjusted and shared adjusted models.
