# Supplementary material for: Personality predictors of dementia diagnosis and neuropathological burden: An individual participant data meta‐analysis
Source: Alzheimers Dement. 2023 Nov 29;20(3):1497–514. doi: 10.1002/alz.13523 (PMC10947984; doi:10.1002/alz.13523)
Supplement: Supplementary file 1 — Supplemental Information. [file ALZ-20-1497-s001.docx]

| **Table S1** *Personality Trait and Subjective Well-Being Measurement Inventories, Scales, and Assessments Across Samples* | | | |
| --- | --- | --- | --- |
| **Measure** | **Source** | **Scale** | **Used (Available)** |
| **EAS** | | | |
| Extraversion | 24 items from the IPIP NEO | 1 "strongly disagree" to 5 "strongly agree" | 2004 (Annual Follow-ups) |
| Agreeableness | 24 items from the IPIP NEO | 1 "strongly disagree" to 5 "strongly agree" | 2004 (Annual Follow-ups) |
| Conscientiousness | 24 items from the IPIP NEO | 1 "strongly disagree" to 5 "strongly agree" | 2004 (Annual Follow-ups) |
| Neuroticism | 24 items from the IPIP NEO | 1 "strongly disagree" to 5 "strongly agree" | 2004 (Annual Follow-ups) |
| Openness to Experience | 24 items from the IPIP NEO | 1 "strongly disagree" to 5 "strongly agree" | 2004 (Annual Follow-ups) |
| Positive Affect | --- | --- | --- |
| Negative Affect | --- | --- | --- |
| Satisfaction with Life | --- | --- | --- |
| **GSOEP** | | | |
| Extraversion | 3 items from the 15 item BFI-S (John, Naumann, & Soto, 2008, and Lang, Lüdtke, & Asendorpf, 2001) | 1 "does not apply at all" to 7 " applies perfectly" | 2005 (2005, 2009, 2013, 2017) |
| Agreeableness | 3 items from the 15 item BFI-S (John, Naumann, & Soto, 2008, and Lang, Lüdtke, & Asendorpf, 2001) | 1 "does not apply at all" to 7 " applies perfectly" | 2005 (2005, 2009, 2013, 2017) |
| Conscientiousness | 3 items from the 15 item BFI-S (John, Naumann, & Soto, 2008, and Lang, Lüdtke, & Asendorpf, 2001) | 1 "does not apply at all" to 7 " applies perfectly" | 2005 (2005, 2009, 2013, 2017) |
| Neuroticism | 3 items from the 15 item BFI-S (John, Naumann, & Soto, 2008, and Lang, Lüdtke, & Asendorpf, 2001) | 1 "does not apply at all" to 7 " applies perfectly" | 2005 (2005, 2009, 2013, 2017) |
| Openness to Experience | 3 items from the 15 item BFI-S (John, Naumann, & Soto, 2008, and Lang, Lüdtke, & Asendorpf, 2001) | 1 "does not apply at all" to 7 " applies perfectly" | 2005 (2005, 2009, 2013, 2017) |
| Positive Affect | 1 item ("Frequency of being happy in the last 4 weeks") | 1 "very seldom" to 5 "very often" | 2007 (2007-2017) |
| Negative Affect | 3 items (angry, sad, worried) | 1 "very seldom" to 5 "very often" | 2007 (2007-2017) |
| Satisfaction with Life | SWLS (Diener, Emmons, Larsen, & Griffin, 1985) | 0 "low" to 10 "high" | 2005 (1984-2017) |
| **HRS** | | | |
| Extraversion | 5 adjectives from a 25 adjective checklist (Lachman & Weaver, 1997) | **1 "a lot" to 4 "not at all" | 2006/8 (2006/8, 2010/12, 2014/16) |
| Agreeableness | 5 adjectives from a 25 adjective checklist (Lachman & Weaver, 1997) | **1 "a lot" to 4 "not at all" | 2006/8 (2006/8, 2010/12, 2014/16) |
| Conscientiousness | 5 adjectives from a 25 adjective checklist (Lachman & Weaver, 1997) | **1 "a lot" to 4 "not at all" | 2006/8 (2006/8, 2010/12, 2014/16) |
| Neuroticism | 5 adjectives from a 25 adjective checklist (Lachman & Weaver, 1997) | **1 "a lot" to 4 "not at all" | 2006/8 (2006/8, 2010/12, 2014/16) |
| Openness to Experience | 5 adjectives from a 25 adjective checklist (Lachman & Weaver, 1997) | **1 "a lot" to 4 "not at all" | 2006/8 (2006/8, 2010/12, 2014/16) |
| Positive Affect | PANAS-X (Watson & Clark, 1994) | *1 "very much" to 5 "not at all" | 2006-2016 |
| Negative Affect | PANAS-X (Watson & Clark, 1994) | *1 "very much" to 5 "not at all" | 2006-2016 |
| Satisfaction with Life | SWLS (Diener, Emmons, Larsen, & Griffin, 1985) | 1 "strongly disagree" to 7 "strongly agree" | 2006/8 (2006/8, 2010/12, 2014/16) |
| **LISS** | | | |
| Extraversion | 10 items from the 50 item IPIP-50 (Goldberg, 1992) | 1 "very inaccurate" to 5 "very accurate" | 2008 (2008-2018) |
| Agreeableness | 10 items from the 50 item IPIP-50 (Goldberg, 1992) | 1 "very inaccurate" to 5 "very accurate" | 2008 (2008-2018) |
| Conscientiousness | 10 items from the 50 item IPIP-50 (Goldberg, 1992) | 1 "very inaccurate" to 5 "very accurate" | 2008 (2008-2018) |
| Neuroticism | 10 items from the 50 item IPIP-50 (Goldberg, 1992) | 1 "very inaccurate" to 5 "very accurate" | 2008 (2008-2018) |
| Openness to Experience | 10 items from the 50 item IPIP-50 (Goldberg, 1992) | 1 "very inaccurate" to 5 "very accurate" | 2008 (2008-2018) |
| Positive Affect | 10 items (e.g. "interested") | 1 "not at all" to 7 "extremely" | 2008 (2008-2018) |
| Negative Affect | 10 items (e.g. "distressed") | 1 "not at all" to 7 "extremely" | 2008 (2008-2018) |
| Satisfaction with Life | SWLS (Diener, Emmons, Larsen, & Griffin, 1985) | 1 "strongly disagree" to 7 "strongly agree" | 2008 (2008-2018) |
| **RUSH-MAP** | | | |
| Extraversion | 6 items from the NEO Five Factor Inventory | 1 "strongly disagree" to 5 "strongly agree" | Baseline (Baseline) |
| Agreeableness | --- | --- | --- |
| Conscientiousness | 12 items from the NEO Five-Factor Inventory (NEO-FFI; Costa & McCrae, 1992) | 1 "strongly disagree" to 5 "strongly agree" | Baseline (Baseline) |
| Neuroticism | 12 items from the NEO Five-Factor Inventory (NEO-FFI; Costa & McCrae, 1992) | 1 "strongly disagree" to 5 "strongly agree" | Baseline (Baseline) |
| Openness to Experience | --- | --- | --- |
| Positive Affect | 1 item "Overall, how happy are you?" | *1 "Very happy" to 4 "Not happy at all" | Baseline (Annual Clinical Followups) |
| Negative Affect | PANAS-X (Watson & Clark, 1994) | 1 "Very slightly or not at al" to 5 "Extremely" | Baseline (Annual Clinical Followups) |
| Satisfaction with Life | SWLS (Diener, Emmons, Larsen, & Griffin, 1985) | *1 "Strongly agree" to 7 "Strongly Disagree" | Baseline (Annual Clinical Followups) |
| **RUSH-ROS** | | | |
| Extraversion | 6 items from the NEO Five Factor Inventory | 1 "strongly disagree" to 5 "strongly agree" | Baseline (Baseline) |
| Agreeableness | 12 items from the NEO Five-Factor Inventory (NEO-FFI; Costa & McCrae, 1992) | 1 "strongly disagree" to 5 "strongly agree" | Baseline (Baseline) |
| Conscientiousness | 12 items from the NEO Five-Factor Inventory (NEO-FFI; Costa & McCrae, 1992) | 1 "strongly disagree" to 5 "strongly agree" | Baseline (Baseline) |
| Neuroticism | 12 items from the NEO Five-Factor Inventory (NEO-FFI; Costa & McCrae, 1992) | 1 "strongly disagree" to 5 "strongly agree" | Baseline (Baseline) |
| Openness to Experience | 12 items from the NEO Five-Factor Inventory (NEO-FFI; Costa & McCrae, 1992) | 1 "strongly disagree" to 5 "strongly agree" | Baseline (Baseline) |
| Positive Affect | 1 item "Overall, how happy are you?" | *1 "Very happy" to 4 "Not happy at all" | Baseline (Annual Clinical Followups) |
| Negative Affect | --- | --- | --- |
| Satisfaction with Life | SWLS (Diener, Emmons, Larsen, & Griffin, 1985) | *1 "Strongly agree" to 7 "Strongly Disagree" | Baseline (Annual Clinical Followups) |
| **SATSA** | | | |
| Extraversion | 9 items from the Eysenck Personality Inventory | **1 "exactly right" to 5 "not right at all" | 1984 (1984, 1987, 1989, 1990, 1992, 1993, 1999, 2002, 2004, 2005, 2007) |
| Agreeableness | 10 items | **1 "exactly right" to 5 "not right at all" | 1984 (1984) |
| Conscientiousness | 10 items | **1 "exactly right" to 5 "not right at all" | 1984 (1984) |
| Neuroticism | 9 items from the Eysenck Personality Inventory | **1 "exactly right" to 5 "not right at all" | 1984 (1984, 1987, 1989, 1990, 1992, 1993, 1999, 2002, 2004, 2005, 2007) |
| Openness to Experience | 25 items from the NEO Personality Inventory | **1 "exactly right" to 5 "not right at all" | 1984 (1984) |
| Positive Affect | 5 items (e.g., "calm", "harmonious") | **1 "exactly right" to 5 "not right at all" | 1984 (1984, 1987, 1989, 1990, 1992, 1993, 1995) |
| Positive Affect | 6 items (e.g., "worried", "tense") | **1 "exactly right" to 5 "not right at all" | 1984 (1984, 1987, 1989, 1990, 1992, 1993, 1995) |
| Satisfaction with Life | 13 items | **1 "exactly right" to 5 "not right at all" | 1984 (1984, 1987, 1989, 1990, 1993, 2004, 2007) |
| **WUSM-MAP** | | | |
| Extraversion | 12 items from the NEO Five-Factor Inventory (NEO-FFI; Costa & McCrae, 1992) | 1 "strongly disagree" to 5 "strongly agree" | Baseline (Clinical Follow-ups) |
| Agreeableness | 12 items from the NEO Five-Factor Inventory (NEO-FFI; Costa & McCrae, 1992) | 1 "strongly disagree" to 5 "strongly agree" | Baseline (Clinical Follow-ups) |
| Conscientiousness | 12 items from the NEO Five-Factor Inventory (NEO-FFI; Costa & McCrae, 1992) | 1 "strongly disagree" to 5 "strongly agree" | Baseline (Clinical Follow-ups) |
| Neuroticism | 12 items from the NEO Five-Factor Inventory (NEO-FFI; Costa & McCrae, 1992) | 1 "strongly disagree" to 5 "strongly agree" | Baseline (Clinical Follow-ups) |
| Openness to Experience | 12 items from the NEO Five-Factor Inventory (NEO-FFI; Costa & McCrae, 1992) | 1 "strongly disagree" to 5 "strongly agree" | Baseline (Clinical Follow-ups) |
| Positive Affect | --- | --- | --- |
| Negative Affect | --- | --- | --- |
| Satisfaction with Life | --- | --- | --- |
