# Supplementary material for: Personality predictors of dementia diagnosis and neuropathological burden: An individual participant data meta‐analysis
Source: Alzheimers Dement. 2023 Nov 29;20(3):1497–514. doi: 10.1002/alz.13523 (PMC10947984; doi:10.1002/alz.13523)
Supplement: Supplementary file 2 — Supplemental Information. [file ALZ-20-1497-s003.docx]

| **Table S2** *List of Measures Across Samples* | | | | | | | | |
| --- | --- | --- | --- | --- | --- | --- | --- | --- |
| **Measure** | **GSOEP** | **HRS** | **LISS** | **SATSA** | **RUSH-MAP** | **RUSH-ROS** | **ADRC-MAP** | **EAS** |
| **Cognitive** | | | | | | | | |
| Extraversion | X | X | X | X | X | X | X | X |
| Agreeableness | X | X | X |  |  | X | X | X |
| Conscientiousness | X | X | X |  | X | X | X | X |
| Neuroticism | X | X | X | X | X | X | X | X |
| Openness to Experience | X | X | X | X |  | X | X | X |
| Satisfaction with Life | X | X | X | X | X | X | X |  |
| Positive Affect | X | X | X |  | X | X |  |  |
| Negative Affect | X | X | X |  | X |  |  |  |
| Self-Reported Dementia | X | X | X | X | X | X | X | X |
| Braak Stage |  |  |  |  | X | X | X | X |
| CERAD |  |  |  |  | X | X | X |  |
| Lewy Body Disease |  |  |  |  | X | X | X | X |
| Gross Cerebral Infarcts |  |  |  |  | X | X | X |  |
| Gross Cerebral Microinfarcts |  |  |  |  | X | X | X |  |
| Cerebral Atherosclerosis |  |  |  |  | X | X | X |  |
| Cerebral Amyloid Angiopathy |  |  |  |  | X | X | X |  |
| **Covariates** | | | | | | | | |
| Arteriolosclerosis |  |  |  |  | X | X | X |  |
| Hippocampal Sclerosis |  |  |  |  | X | X | X | X |
| Block Design |  |  |  | X |  |  | X | X |
| Digits Forward |  |  |  | X | X | X | X | X |
| Digits Backward |  | X |  | X | X | X | X | X |
| Information |  |  |  | X |  |  | X | X |
| Digit Symbol | X |  |  | X | X | X | X |  |
| Cued Recall |  |  |  |  | X | X | X |  |
| Free Recall |  | X |  |  | X | X | X | X |
| Category Fluency | X |  |  |  | X | X | X | X |
| Picture Memory |  |  |  | X |  |  |  |  |
| Figure Logic |  |  |  | X |  |  |  |  |
| Vocabulary | X | X |  |  |  |  |  |  |
| Boston Naming Test |  |  |  | X |  |  |  | X |
| **Outcomes** | | | | | | | | |
| Progressive Matrices |  |  |  | X |  |  |  |  |
| Serial 7's |  | X |  |  |  |  |  |  |
| Trail-Making Task |  |  |  |  |  |  | X | X |
| Card Rotation |  |  |  |  |  |  |  |  |
| Age | X | X | X | X | X | X | X | X |
| Gender | X | X | X | X | X | X | X | X |
| Education | X | X | X | X | X | X | X | X |
| Race |  |  |  |  | X | X | X | X |
| Ethnicity |  |  |  |  | X | X | X | X |
| Marital Status | X | X | X | X | X | X | X | X |
| **Personality** | | | | | | | | |
| Self-Rated Health | X | X | X | X |  |  | X | X |
| Heart Problems | X | X | X | X | X | X | X | X |
| Stroke | X | X | X | X | X | X | X | X |
| Diabetes | X | X | X | X | X | X | X | X |
| Cancer | X | X | X | X | X | X | X | X |
| Respiratory Problems | X | X | X | X | X | X | X | X |
| Smoking | X | X | X | X | X | X |  | X |
| Alcohol | X | X | X | X | X | X |  | X |
