# Supplementary material for: Personality predictors of dementia diagnosis and neuropathological burden: An individual participant data meta‐analysis
Source: Alzheimers Dement. 2023 Nov 29;20(3):1497–514. doi: 10.1002/alz.13523 (PMC10947984; doi:10.1002/alz.13523)
Supplement: Supplementary file 3 — Supplemental Information. [file ALZ-20-1497-s004.docx]

| **Table S3** *List of Prior Publications Examining Personality-Dementia or Neuropathology Associations* | | | | | | | | | |
| --- | --- | --- | --- | --- | --- | --- | --- | --- | --- |
| **Paper** | **Year** | **Ref #** | **Sample(s)** | **N** | **Data** | **Measures** | **Outcomes** | **Findings** | **Comparison with Current Study** |
| Terracciano et al. | 2014 | 5 | BLSA (IPD) | N = 1671 | IPD + meta-analysis | E, A, C, N, O | Cognitive Status | N -> higher risk C -> lower risk | BLSA not used in the present sample. Meta-analyzed results from Rush-MAP and ROS, but included much smaller sample (we used an additional 10-15 years of data). |
| Wilson et al. | 2006 | 50 | Rush-MAP | N = 648 | IPD | N | Cognitive Status | N -> higher risk | Additional waves of data (~15 years of additional follow-ups).  Used Cox Proportional Hazards Models.  Only examined Neuroticism |
| Wilson et al. | 2007 | 6 | ROS | N = 997 | IPD | C | Cognitive Status | C -> lower risk | Additional waves of data (~15 years of additional follow-ups).  Used Cox Proportional Hazards Models.  Only examined Conscientiousness |
| Wilson et al. | 2015 | -- | ROS  Rush-MAP | N = 309 | IPD | C | NFT, Lewy bodies, chronic gross cerebral infarctions, and hippocampal sclerosis, terminal decline | C -> slower terminal but not preterminal decline | Additional waves of data. Focus was on cognitive decline, not diagnosis. Only examined deceased participants. Additional waves of follow-up data. |
| Terracciano et al. | 2017 | 49 | HRS | N = 13,882 | IPD | E, A, C, N, O | Cognitive Status | N -> higher risk C, A -> lower risk | Used Cox Proportional Hazards Models. Additional waves of follow-up data. |
| Yoneda et al. | 2020 | 52 | EAS  LASA | N (EAS) = 785 N (LASA) = 1300 | IPD | E, A, C, N, O | Cognitive Status | N increases -> higher risk | We additionally include neuropathology data from EAS. Examined associations between personality and change and cognitive status, not baseline levels. |
| Duchek et al. | 2020 | 34 | WUSM-MAP | N = 436 | IPD | N, C | In vivo neuropathology Clinical Dementia Ratings | C -> lower early dementia risk | Focused on in vivo neuropathology, rather than neuropathology at autopsy. Only examined taransitions to early stage dementia. Only investigated N and C. Additional waves of follow-up data. |
| Graham et al. | 2021a | 51 | ROS  Rush-MAP | N (ROS) = 783 N (MAP) = 857 | IPD | E, A (ROS), C, O (ROS), N | Cognitive Resilience (residual of global cognitive function / decline regressed on pathology) | N-> worse resilience | Focused on association between personality traits and asymmetry between neuropathology and cognitive function / decline.  Did not account for dementia diagnoses.  Additional waves of follow-up data. |
| Aschwanden et al. | 2020 | -- | ELSA  HILDA | N (ELSA) = 6,887 N (HILDA) = 2,778 | Meta-analysis | E, A, C, N, O | Cognitive Status from cognitive tests | C -> lower dementia risk (ELSA only) | Used Cox Proportional Hazards Models Different cognitive status indicator that allowed us to use more of the sample. Additional waves of follow-up data. |
| Aschwanden et al. | 2021 | 19 | Rush-MAP  ROS  WUSM-MAP  ELSA  HILDA | N (Rush-MAP) = 648 N (ROS) = 904 N (WUSM-MAP) = 436 N (ELSA) = 6887 N (HILDA) = 2778 | IPD | E, A, C, N, O | Cognitive Status | N -> higher risk C -> lower risk | Used Cox Proportional Hazards Models. Only meta-analyzed existing previous data that, in many cases, had many fewer waves of follow-up. Covariates determined by previous publications. |
| Graham et al. | 2021b | 22 | EAS  Rush-MAP  ROS  SATSA | N (EAS) = 737 N (Rush-MAP) = 1233 N (ROS) = 1466 N (SATSA) = 707 | IPD | E, A, C, N, O | Cognitive Status | O -> post-dementia decline | Additional waves of data for EAS, Rush-MAP, and ROS. Focus was on personality predictors of cognitive decline (slope) and cognitive decline following dementia diagnosis. No reported associations between personality traits and cognitive status. |
